# A SYSTEMATIC REVIEW OF MEASURES USED IN STUDIES LINKING SOCIAL ISOLATION, LONELINESS, AND PHYSICAL HEALTH

**DOI:** 10.1093/geroni/igae098.3223

**Published:** 2024-12-31

**Authors:** Priya Nambisan, Sushma Gangula, Julie Ellis, Murad Taani, Yura Lee

**Affiliations:** University of Wisconsin Milwaukee, Milwaukee, Wisconsin, United States; University of Wisconsin Milwaukee, Milwaukee, Wisconsin, United States; University of Wisconsin Milwaukee, Milwaukee, Wisconsin, United States; University of Wisconsin Milwaukee, Milwaukee, Wisconsin, United States; University of Wisconsin-Milwaukee, Milwaukee, Wisconsin, United States

## Abstract

Social isolation (SI) and loneliness significantly impact public health, particularly among older adults, leading to adverse physical health outcomes. Despite extensive research in this field, there is considerable variability in how these factors are measured. Our systematic review, following the PRISMA method, identified 22 relevant studies. Among these, 18 primarily utilized secondary data and assessed social isolation through questions covering marital status, contact frequency with family, friends, and participation in social activities. Composite indices and scales were often constructed from these measures, with scores indicating low/high social isolation. However, it’s essential to note that loneliness, which can be felt despite social interaction, requires validated scales for accurate measurement. Out of the 22 studies, 4 didn’t employ validated scales for either SI or loneliness, while 6 lacked validation for SI, and 5 out of 20 for loneliness. Measurement scales varied for loneliness, from simple classifications to Likert scales, with higher scores indicating heightened loneliness. Some studies adopted established scales like the Bude and Lantermann scale, while others employed modified versions or analogues. Our analysis highlights the importance of using validated measures to assess these variables accurately. Markedly, different measurement approaches yielded distinct impacts on health outcomes. Moving forward, standardization and validation of measurement tools are critical for enhancing the reliability and comparability of research findings in this area.

